# Circular RNA ITCH has inhibitory effect on ESCC by suppressing the Wnt/β-catenin pathway

**DOI:** 10.18632/oncotarget.3469

**Published:** 2015-02-28

**Authors:** Fang Li, Liyuan Zhang, Wei Li, Jieqiong Deng, Jian Zheng, Mingxing An, Jiachun Lu, Yifeng Zhou

**Affiliations:** ^1^ Department of Genetics, Medical College of Soochow University, Suzhou, China; ^2^ Department of Radiotherapy & Oncology, The Second Affiliated Hospital of Soochow University, Suzhou, China; ^3^ The Institute for Chemical Carcinogenesis, The State Key Lab of Respiratory Disease, Guangzhou Medical University, Guangzhou, China

**Keywords:** Cir-ITCH, ESCC, Wnt/β-catenin pathway

## Abstract

Circular RNAs with exonic sequences represent a special form of non-coding RNAs, discovered by analyzing a handful of transcribed genes. It has been observed that circular RNAs function as microRNA sponges. In the present study, we investigated whether the expression of circular RNAs is altered during the development of esophageal squamous cell carcinoma (ESCC). Using a TaqMan-based reverse transcriptase polymerase chain reaction assay, the relationship between *cir-ITCH* and ESCC was analyzed in a total of 684 ESCC and paired adjacent non-tumor tissue samples from eastern and southern China. We found that *cir-ITCH* expression was usually low in ESCC compared to the peritumoral tissue. The functional relevance of *cir-ITCH* was further examined by biochemical assays. As sponge of miR-7, miR-17, and miR-214, *cir-ITCH* might increase the level of *ITCH*. *ITCH* hyper expression promotes ubiquitination and degradation of phosphorylated Dvl2, thereby inhibiting the Wnt/β-catenin pathway. These results indicate that *cir-ITCH* may have an inhibitory effect on ESCC by regulating the Wnt pathway.

## INTRODUCTION

Esophageal cancer is the eighth most common cancer worldwide and sixth most common cause of cancer death [1]. One of the main subtypes is esophageal squamous cell carcinoma (ESCC), which is a malignancy that arises from esophageal epithelial cells [2]. Cancer is widely regarded as a genetic disease, and ESCC is no exception, but the molecular and genetic basis of esophageal carcinogenesis remains largely unknown [3, 4].

High-throughput RNA sequencing (RNA-Seq), an emerging method to study the RNA regulation mechanism in the whole genome, has been able to detect circular RNA [5]. Circular RNA, in general does not encode protein, but can occur in any genomic region; 85% of circular RNAs are aligned in sense orientation to known protein-coding genes, and they span 1–5 exons [6]. The existence of circular RNA was proposed for several years in early research, for example, the circular testis-determining gene, *SRY* [7]. The most well-known circular RNA is *CDR1*, coding for cerebellar degeneration-related protein 1, which has been observed in all domains of life, but overall, circular RNAs are considered extremely rare in nature [8]. Recently, circular RNAs were proposed to harbor microRNAs (miRNAs), and were found to be enriched with functional miRNA binding sites [6, 9]. Mature miRNAs always play an important regulatory role in cell growth, proliferation, differentiation, and cell death. Following database analysis of the study by Memczak et al. on circular RNA, we found that *cir-ITCH* spanned several exons of the E3 ubiquitin (Ub) protein ligase (ITCH). Moreover, both *cir-ITCH* and the 3′-untranslated region (UTR) of *ITCH* shared some miRNAs binding sites, which might suggest its role as a miRNA sponge [6, 10]. ITCH belongs to the Nedd4-like E3 family and typically contains 4 WW domains known to associate with PPxY-containing targets [11]. The targets of *ITCH* are usually associated with tumor formation and chemosensitivity [12]. A study has shown that ITCH degrades the phosphorylated form of disheveled (Dvl) via the proteasome pathway, thus inhibiting the action of the canonical Wnt pathway [13]. The Wnt/β-catenin pathway plays a role in the carcinogenesis of many cancer subtypes including hepatocellular carcinoma, pancreatic cancer, ovarian carcinoma, and ESCC [14-17]. A previous study has demonstrated that circular RNA has anti-cancer effects in malignant melanoma cell lines [18]. However, there are no reported studies on the functional roles of circular RNA in ESCC.

In this study, we hypothesized that *cir-ITCH* might influence the expression level of *ITCH* and may be involved in ESCC development. To address this hypothesis, we conducted this study to delineate any *cir-ITCH* transcriptional aberrations between ESCC and paired adjacent non-neoplastic tissues obtained from two distinct eastern and southern Chinese centers.

## RESULTS

### Identification of *cir-ITCH*

**Figure 1:**
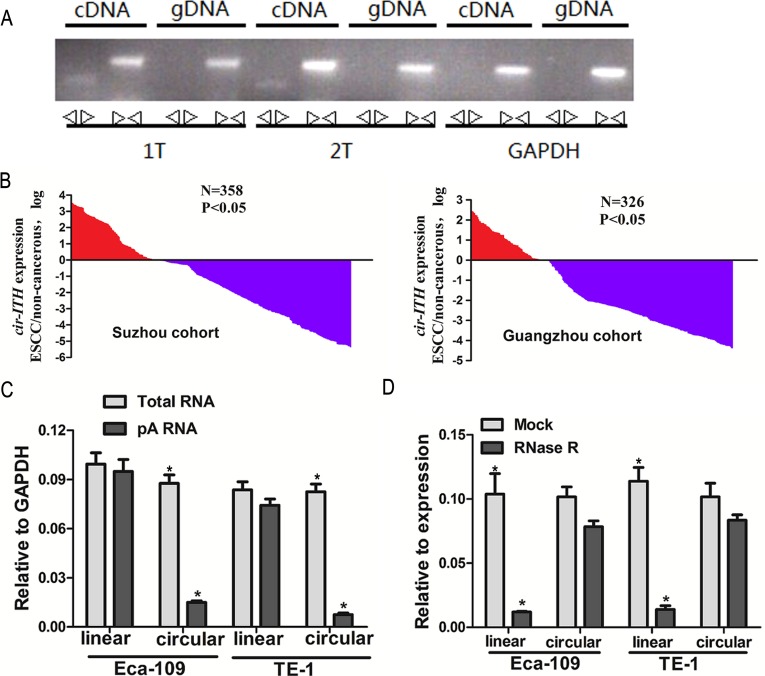
*Cir-ITCH* expression level is strongly associated with ESCC (A) Divergent primers amplify circular RNAs in cDNA but not genomic DNA (gDNA). Convergent primers can amplify both circular RNAs and linear RNAs, GAPDH, linear control. (B) The *cir-ITCH* was expressed at a higher level in approximately 70.1% (251/358patients in the Suzhou cohort) and 71.2% (232/326 patients in the Guangzhou cohort) of the ESCC adjacent tissues compared to matched ESCC tissues. The expression level of *cir-ITCH* was analyzed by qRT-PCR based on Taq-man and normalized to *GAPDH*. Data are represented as mean±SEM from three independent experiments. (C) Random primers and oligodT primers were used respectively in the reverse transcription experiments. The predicted circular RNA is absent in poly (A) enriched samples. (D) The predicted circular RNA is resistant to RNase R treatment.

RT-PCR assays were used in this study to verify the circular form of *ITCH*. We designed two sets of primers for *ITCH*: a divergent set that was expected to amplify only the circular form, and an opposite-directed set to amplify the linear forms. Our results showed that the circular form was amplified using the divergent primers (Figure 1A). cDNA and genomic DNA were used as templates, and as expected, no amplification was observed with the divergent primers on genomic DNA. *GAPDH* was used as a linear control (Figure 1A).

### Expression of *cir-ITCH* in ESCC tissues

A TaqMan-based qRT-PCR assay was used for the divergent primer set to determine the levels of *ITCH* in 358 and 326 paired ESCC samples and matched non-cancerous tissues from eastern (Suzhou) and southern Chinese centers (Guangzhou), respectively, *cir-ITCH* was expressed at a higher level in approximately 70.1% (251/358 patients in the Suzhou cohort) and 71.2% (232/326 patients in the Guangzhou cohort) of the ESCC adjacent tissues compared to matched ESCC tissues (Figure 1B).

### Characterization of *cir-ITCH* in ESCC cells

To study circular RNA at the cellular level, and to test the cyclization mechanism of RNA, we constructed a vector-based system expressing *cir-ITCH*. The constructed plasmids were transiently transfected into Eca-109 and TE-1 cells.

Random and oligo-dT primers were used in the reverse transcription experiments. We expected that circular products would be depleted in the poly-(A)-enriched samples, in contrast to the linear products. When the oligo-dT primers were used, the relative expression of linear *ITCH* was significantly higher than that of circular *ITCH* (5.8-fold in Eca-109 cells and 10-fold in TE-1 cells; Figure 1C) [18].

To confirm further the circular characteristics of *cir-ITCH*, we used the enzyme RNase R, a highly processive 3′ to 5′ exoribonuclease that does not act on circular RNAs [19, 20]. As expected, in contrast to the linear control RNAs (10-fold in Eca-109 cells and 11-fold in TE-1 cells), the circular RNA was resistant to RNase R treatment (Figure 1D).

### *cir-ITCH* interacts with miRNAs

**Figure 2:**
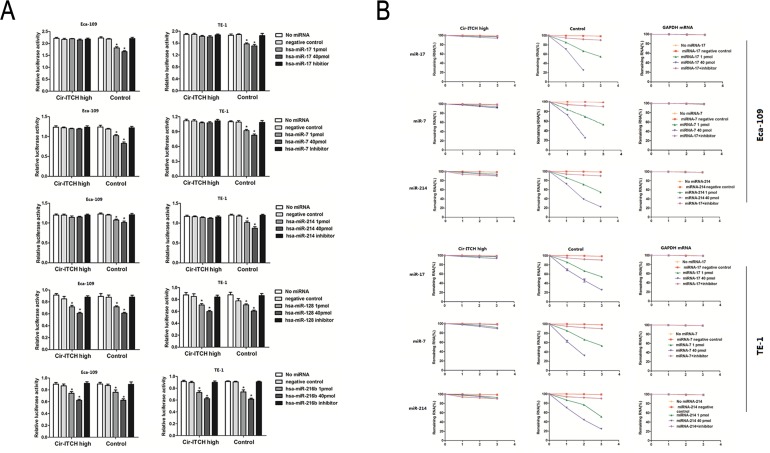
*Cir-ITCH* is functioning as microRNA sponges (A) Relative luciferase activity of the psiCHECK-2-ITCH constructs co-transfected with miR-17, miR-7, miR-214, miR-216b and miR-128 and inhibitor in Eca-109 and TE-1cells. In *Cir-ITCH* hyper-expression cells, there were no significant differences in luciferase activity when psiCHECK-2-*ITCH*-binding site with miRNAs were cotransfected into Eca-109 and TE-1cells. Six replicates for each group and the experiment repeated at least three times. Data are mean±SEM. (B) Eca-109 and TE-1 cells after transfected with *Cir-ITCH* and Control lentiviruses were respectively transfected with miR-17,miR-7,miR-214 and inhibitor for 24 h and were then further exposed to actinomycin D for 1, 2 and 3 h. Cells were harvested and the stability of *cir-ITCH* mRNA was analyzed by qRT-PCR relative to time 0 after blocking new RNA synthesis with actinomycin D; data are mean±SEM, normalized to *GAPDH.*

miRanda (http://www.microrna.org/) and TargetScan/TargetScanS (http://www.targetscan.org/) software were used to predict the binding sites for human microRNA within *cir-ITCH* and the 3′-UTR region of *ITCH*. The results of both showed that miR-216b, miR-17, miR-214, miR-7, and miR-128 could bind to the 3′-UTR of *ITCH* and *cir-ITCH*. The sequence of the predicted 5 microRNAs binding sites were presented in Supplementary Table 1. The 5 miRNAs and one of the reporter constructs were transiently co-transfected into ESCC cells, and subsequently, luciferase activity was assessed. The results of the empty vector construct and miR-17 showed that luciferase activity was significantly reduced in a concentration-dependent manner in Eca-109 cells (Figure 2A), and this trend was also present with mimics of miR-7, miR-214, miR-216b, and miR-128 in the control cells (1 pmol miRNA-17: 1.817 ± 0.022 versus 2.187 ± 0.009, *P* = 0.02; 40 pmol miRNA-17: 1.656 ± 0.02 versus 2.187 ± 0.009, *P* = 0.005; 1 pmol miRNA-7: 1.019 ± 0.014 versus 1.187 ± 0.009, *P* = 0.04; 40 pmol miRNA-7: 0.828 ± 0.016 versus 1.187 ± 0.009, *P* = 0.007; 1 pmol miRNA-214: 1.073 ± 0.014 versus 1.213 ± 0.012, *P* = 0.02; 40 pmol miRNA-214: 1.015 ± 0.02 versus 1.213 ± 0.012, *P* = 0.004; 1 pmol miRNA-216b: 0.742 ± 0.014 versus 0.873 ± 0.012, *P* = 0.005; 40 pmol miRNA-216b: 0.623 ± 0.009 versus 0.873 ± 0.012, *P* = 0.001; 1 pmol miRNA-128: 0.715 ± 0.01 versus 0.852 ± 0.02, *P* = 0.02; 40 pmol miRNA-128: 0.610 ± 0.006 versus 0.852 ± 0.02, *P* = 0.012). However, in cells with *cir-ITCH* hyperexpression, the results showed that there were no significant differences in luciferase activity when the psiCHECK-2-*ITCH*-binding-site vector and miRNAs were co-transfected into Eca-109 cells except the miR-216b and miR-128 (1 pmol miRNA-17: 2.196 ± 0.007 versus 2.215 ± 0.02, *P* = 0.68; 40 pmol miRNA-17: 2.145 ± 0.02 versus 2.215 ± 0.02, *P* = 0.2; 1 pmol miRNA-7: 1.197 ± 0.006 versus 1.22 ± 0.01, *P* = 0.41; 40 pmol miRNA-7: 1.190 ± 0.007 versus 1.22 ± 0.01, *P* = 0.13; 1 pmol miRNA-214: 1.157 ± 0.011 versus 1.191 ± 0.006, *P* = 0.206; 40 pmol miRNA-214: 1.151 ± 0.008 versus 1.191 ± 0.006, *P* = 0.264; 1 pmol miRNA-216b: 0.741 ± 0.014 versus 0.897 ± 0.013, *P* = 0.0005; 40 pmol miRNA-216b: 0.623 ±0.009 versus 0.897 ± 0.013, *P* = 0.001; 1 pmol miRNA-128:0.717 ± 0.013 versus 0.914 ± 0.013, *P* = 0.00009; 40 pmol miRNA-128: 0.610 ± 0.008 versus 0.914 ± 0.013, *P*=0.002). Similar results were obtained in TE-1 cells (Figure 2A).

We next investigated *cir-ITCH* stability. Eca-109 and TE-1 cells transfected with the plasmid construct were treated with actinomycin D, a transcription inhibitor, in the presence of 1 or 40 pmol of miR-17, miR-7, and miR-214, and total RNA was harvested at indicated time points. There was little change in *cir-ITCH* levels in Eca-109 cells following incubation with actinomycin D for 1–3 hours, while the associated control remained level at 20–30%; the former *cir-ITCH* levels were significantly higher than the latter (*P* < 0.01). We repeated these experiments in TE-1 cells with the same results (Figure 2B).

### Correlation analysis of *cir-ITCH* and *ITCH* in ESCC

**Figure 3:**
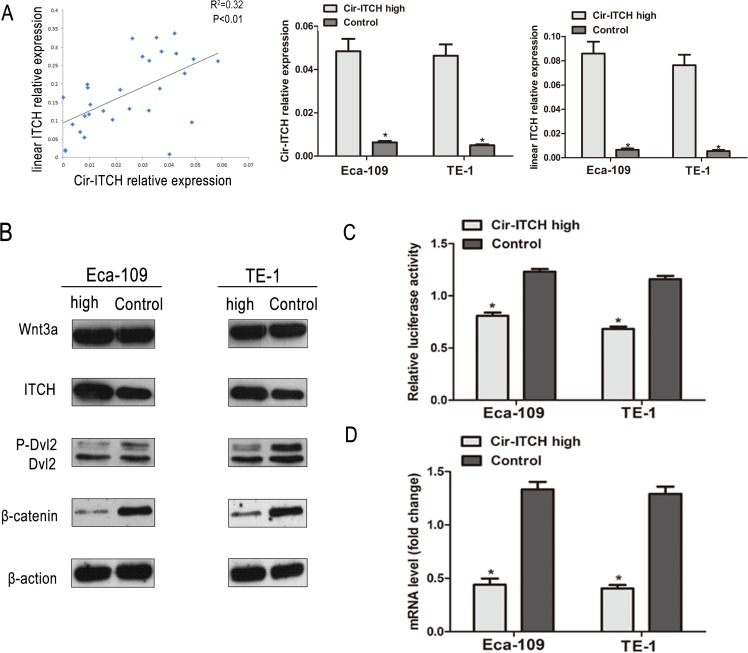
*Cir-ITCH* involves in the regulation Wnt Signaling *in vivo* (A) The linear correlations between the *cir-ITCH* expression levels and linear *ITCH* were observed. The relative expression value was normalized by *GAPDH* expression level. (B) The protein levels of ITCH, Wnt3a, Dvl2 and β-catenin was assessed in ESCC cells (Eca-109 cells and TE-1 cells) by Western blot after *cir-ITCH* or Control lentiviruses infection. β *-actin* was loading control. Data was representative of three independent experiments. (C) A TCF luciferase reporter assay was performed using phRL-TK and TOPFlash (the wild-type TCF reporter). The luciferase activity was normalized to the Renilla luciferase activity. (D) The mRNA level of c-MYC was detected by quantitative RT-PCR after transfected with *cir-ITCH* or Control lentiviruses in ESCC cells. Data are mean±SEM and representative of three independent experiments.

We evaluated the expression of *cir-ITCH* in a randomly selected cohort of 30 pairs of ESCC adjuvant non-cancerous tissues from Suzhou. The results showed that patients with higher *cir-ITCH* expression levels in ESCC tissues had a substantial up-regulation of linear *ITCH.* Furthermore, we tested the mRNA expression levels of linear *ITCH* in constructed *cir- ITCH* cells with stable high expression, and the results showed that the linear *ITCH* positively correlated with levels of *cir- ITCH* (R^2^ = 0.32, *P* < 0.01; Figure 3A).

### *cir-ITCH* is involved in the regulation of Wnt Signaling *in vivo*

Previous studies using the yeast two-hybrid system and co-immunoprecipitation assays have shown that ITCH is a novel Dvl-interacting protein. The ITCH protein can ubiquitinate the phosphorylated form of Dvl and promote its degradation, thereby inhibiting canonical Wnt signaling [21]. To confirm whether *cir-ITCH* regulates the Wnt/β-catenin signaling pathway in ESCC cells, we used a β-catenin/T-cell factor (TCF)-responsive luciferase reporter assay. In Eca-109 cells, we used an anti-Dvl2 antibody to test endogenous Dvl2 levels. Transient *ITCH* hyperexpression reduced the intensity of Dvl2, but the effect was not so obvious in the control cells. A western blot analysis was performed to determine β-catenin levels in cells with *ITCH* hyperexpression, and as shown in Figure 3B, there was an obvious decrease in β-catenin levels. We overexpressed *ITCH* in Eca-109 cells and evaluated its effects using the TOP-Flash reporter system. As shown in Figure 3C, overexpression of *ITCH* significantly suppressed relative TCF transcriptional activity. Then, we investigated the effect of *ITCH* on the expression of endogenous Wnt target genes like *c-Myc*, in cells transfected with *cir-ITCH*, and discovered that the Wnt target genes were further suppressed (Figure 3D).

### *cir-ITCH* regulates the cell cycle of ESCC cells

**Figure 4:**
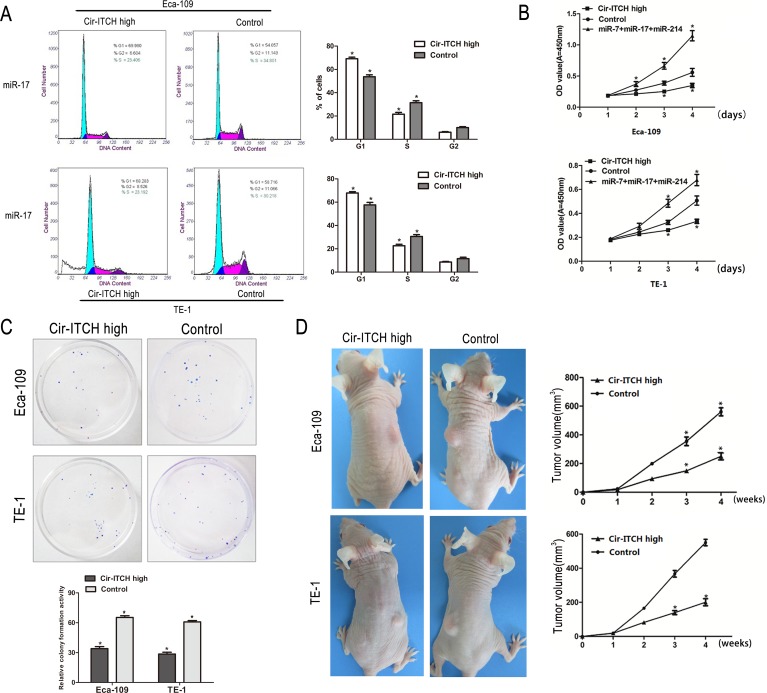
The effects of ectopic*Cir-ITCH* expression on ESCC cell cycle and proliferation (A) Cell cycle analysis of Eca-109 and TE-1 cells after transfected with *Cir-ITCH*, or Control lentiviruses. Flow cytometry data are represented as mean±SEM and are based on three independent experiments. (B) Eca-109 and TE-1 cells were seeded in 96-well plates after transfected with *Cir-ITCH* and Control lentiviruses, and cell proliferation was performed daily for 3 days using the CCK-8 assay. Six replicates for each group and the experiment repeated three times. Data are mean±SEM. *P<0.05 compared with controls. (C) Representative colony formation assay in Eca-109 and TE-1 cells after transfected with *Cir-ITCH* and Control lentiviruses, the numbers of colonies in vector-transfected controls were set to 100%. Data are present as mean±SEM from three experiments. (D) Representative images of xenografts in each group 2 weeks after subcutaneously implanted ESCC cells stably expressing *Cir-ITCH* and respective empty vector (left). Mean tumor volumes from eight nude mice of each group are shown at different time points (right). *P<0.05 compared with controls.

We investigated the effects of *cir-ITCH* on cell cycle using fluorescence-activated cell sorting (FACS) analysis of PI-stained ESCC cells. The representative results of the cell cycle distribution in empty vector and *cir-ITCH* transfected Eca-109, and TE-1 cells in the presence of miR-17 are shown in (Figure 4A). It is evident that overexpression of *cir-ITCH* resulted in a statistically significant accumulation of ESCC cells in the G1 phase (Eca-109 cells: 14.4% increase, *P* = 0.007; TE-1 cells: 9.6% increase, *P* = 0.007) accompanied by a decrease in the S phase (Eca-109 cells: 9.9% decrease, *P* = 0.002; TE-1 cells: 7.2% decrease, *P* = 0.026), compared with the controls. The cell cycle results for miR-7 and miR-214 are shown in Supplementary Figure 1A, 1B.

### *cir-ITCH* modulates cell growth

We performed colony formation and CCK-8 assays to test the effects of *cir-ITCH* on ESCC cell proliferation. Supporting our overexpression data, CCK-8 assay results revealed that after 3 days of culture in *cir-ITCH-*overexpressing Eca-109 and TE-1 cells in the presence of miR-17, miR-7, and miR-214, cell proliferation dramatically decreased (Eca-109 cells: 20% decrease; TE-1 cells: 17.4% decrease) compared to the controls (Figure 4B). CCK-8 experiment results with miR-17, miR-7, and miR-214 showed the same trend (Supplementary Figure 1C).

Consistently, colony formation assays performed at 2 weeks showed that cells with *cir-ITCH-*overexpression had reduced colony-forming ability compared to the control groups (Eca-109 cells: 31.25% decrease; TE-1 cells: 32.25% decrease; Figure 4C). Thus, both assays show that *cir-ITCH* modulates cell proliferation.

### *cir-ITCH* suppresses tumor growth

As shown in (Figure 4D), Eca-109 and TE-1 cells with up-regulated *cir-ITCH* (Eca-109-cir-*ITCH* and TE-1-cir-*ITCH* cells) were subcutaneously injected into the back flank of nude mice.

Our results showed that the growth of tumors from up-regulated *cir-ITCH* xenografts was significantly inhibited compared with that of the control xenografts: 250.6 ± 21.6 mm^3^ versus 560.7.0 ± 24.5 mm^3^ for Eca-109 cells (*P* < 0.001); and 200.4 ± 17.9 mm^3^ versus 550.2 ± 16.3 mm^3^ for TE-1 cells (*P* < 0.001), respectively.

## DISCUSSION

Through a series of functional experiments, we identified a *cir-ITCH* circular RNA whose role had not yet been elucidated. Here, we demonstrated that *cir-ITCH* acts as a miRNA sponge, increases the level of *ITCH,* and provokes ubiquitin-mediated Dvl2 degradation, which inhibits canonical Wnt signaling. These results illustrate the basic interaction between circular RNA, miRNA, and protein in cells, and alteration of this fine regulation may contribute to cancer initiation and progression.

*Cir-ITCH* is located on chromosome 20q11.22 on the plus strand, is aligned in a sense orientation to the known protein-coding gene *ITCH,* and spans exons 6–13. ITCH is a member of the E3 ubiquitin ligases that regulate protein stability and immunological responses, as well as cancer progression [22]. ITCH was initially identified when disruption of the *ITCH* gene induced a fatal autoimmune inflammatory condition [23]. Latterly, ITCH was found to be crucial in the control of proteasome degradation of several important substrates such as p63, p73, Notch1, and Dvl2 [21, 24-26]; all of which are usually associated with tumor formation and chemosensitivity.

The protein-coding function of messenger RNAs can be suppressed by the binding of short miRNA sequences, or miRNAs can guide the effector protein Argonaute to the mRNAs of coding genes to repress their protein production [27-29]. Previous experiments using ectopic expression of miRNA indicated that it could bind to the 3′-UTR of *ITCH* to decrease its expression [30]. Memczak et al. (2013) and Hansen et al. (2011) proposed that *CDR1*, a circular RNA, harbored a miR-7 binding site. This striking feature suggested that circular RNA possibly functioned as a miRNA sponge [6, 9, 31]. In our study, we found that the *cir-ITCH* harbors many miRNA binding sites that could bind to the 3′-UTR of *ITCH*, including those for miR-7, miR-17, and miR-214. Furthermore, these miRNAs have often been associated with cancer. Elevated miR-7 expression has been described in a variety of tumor types including ESCC and has been implicated in oncogenesis, classification, and cancer progression [32, 33]. miR-214 is aberrantly expressed in several human tumors, for example, it is upregulated in ovarian cancer and gastric cancer [34, 35]. miR-216b is a tumor suppressor in nasopharyngeal cancer [36]. miR-17, which is frequently up-regulated in ESCC, has been widely studied, and it is a potential prognostic biomarker for ESCC [37]. Collectively, *cir-ITCH* is involved in regulating miRNAs that are associated with cancer.

*cir-ITCH* acts as a miRNA sponge and increases the level of *ITCH*. ITCH plays an important role in the Wnt/β-catenin pathway and its activation is thought to contribute to the development of some human cancers [38, 39]. Initially, it was reported that activated Wnt/β-catenin signaling was defined by β-catenin nuclear expression and associated with breast cancer [40]. Recent studies have identified that Wnt/β-catenin signaling activation is found in hepatocellular carcinoma [16]. The Wnt/β-catenin pathway has been very well described, as it is important in cancer research. It is mediated by Dvl2, which functions as an essential scaffold protein, bridging the receptors and downstream signaling components [41, 42]. Phosphorylation of Dvl2 is required for Wnt signaling, and is crucial to regulate its stability and activity for proper signal transduction. According to previous research, ITCH could promote the ubiquitination and degradation of phosphorylated Dvl2, and therefore, inhibit canonical Wnt signaling [21]. Our data demonstrated that overexpressed *cir-ITCH* was able to suppress phosphorylated Dvl2 and inhibit Wnt/β-catenin signaling in ESCC cells. The β-catenin/TCF-responsive luciferase reporter assay was used to examine whether a single gene regulates the Wnt/β-catenin signaling pathway. In our study, overexpression of *cir-ITCH* significantly suppressed relative TCF transcriptional activity.

**Figure 5:**
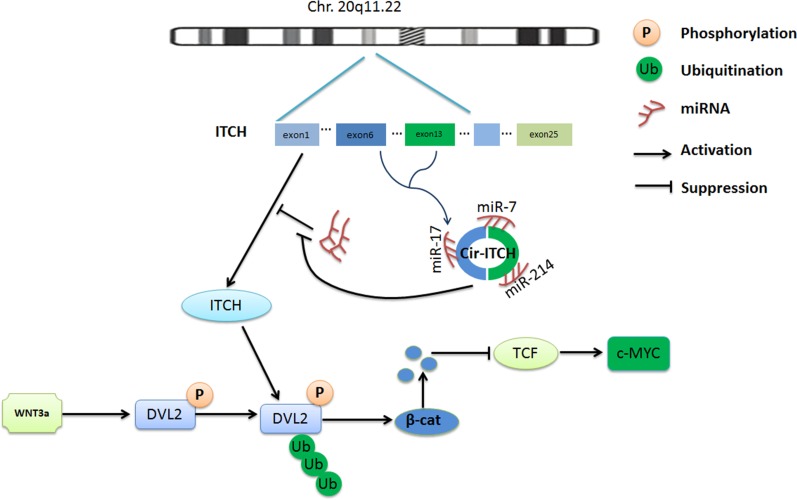
Schematic representation of the method used for the analysis of *Cir-ITCH* regulated progress of ESCC *Cir-ITCH* is a potential tumor suppressor acting through the control of miRNAs activity such as miR-7, miR-17, miR-214 and increase the concentration of *ITCH*, resulting in suppression of the canonical Wnt pathway by degradation of phosphorylated Dvl2, and inhibited the oncogene *c-myc* expression therefore inhibit canonical Wnt signaling.

The oncogene *c-Myc* is a target gene in canonical Wnt signaling; it is frequently overexpressed in many cancers and has a crucial role in cell growth, reproduction, apoptosis, and differentiation [43]. Knockdown of *c-Myc* leads to cell-cycle arrest and limits malignant cancer cell growth. Our cell-cycle results showed the same trend and indicated that *cir-ITCH* affects the expression of *c-Myc*. Therefore, we suggest that *cir-ITCH* may have an antitumor function in ESCC and suppress the canonical Wnt pathway. Based on these findings, we are able to conclude that *cir-ITCH* has an antitumor role by controlling miRNA activity, which increases the concentration of *ITCH*, and results in suppression of the canonical Wnt pathway by degradation of phosphorylated Dvl2, and inhibition of *c-Myc* expression, which may prevent oncogenesis (Figure 5).

In summary, the current study represents an analysis of 2-stage clinical ESCC samples for the circular RNA, *cir-ITCH*. The results show that there is low expression of *cir-ITCH* in ESCC tumor tissues, and that *cir-ITCH* reduces cell viability, and arrests proliferation in ESCC cells. The inhibition effects of *cir-ITCH* may be related to its cooperation with Dvl2 to suppress the Wnt/β-catenin pathway. Overall, our data support the assumption that *cir-ITCH* acts as a miRNA sponge and increases expression of the miRNA target gene *ITCH*. The availability of circularizing the miRNA sponge in cells is a candidate for new strategies for RNA-based cancer diagnosis and therapy.

## MATERIALS AND METHODS

### Study subjects

All subjects in this study were homogenous Han Chinese from eastern or southern China. At the eastern Chinese center, 358 ESCC and corresponding paracancerous tissue samples were obtained from patients at affiliate hospitals of Soochow University (Suzhou). At the southern Chinese center, a further 326 ESCC tissues were harvested from patients at the cancer hospitals affiliated with Guangzhou Medical University. There were no restrictions on age, stage of ESCC, sex or histology. This study was approved by the Medical Ethics Committee of Soochow University and Guangzhou Medical University. The clinical characteristics of all patients are listed in Table 1.

**Table 1:** Baseline demographic and clinical characteristics of study populations

Characteristics	Suzhou population		Guangzhou population		Overall	
	**N**	**(%)**	**N**	**(%)**	**N**	**(%)**
**Age(years)**						
≤40	41	(11.5)	42	(12.9)	83	(12.1)
40-60	203	(56.6)	187	(57.3)	390	(57.0)
≥60	114	(31.9)	97	(29.8)	211	(30.9)
**Sex**						
Male	281	(78.5)	271	(83.1)	552	(80.7)
Female	77	(21.5)	55	(16.9)	132	(19.3)
**Body mass index**						
≤20	79	(22.1)	59	(18.1)	138	(20.2)
20-28	255	(71.2)	245	(75.2)	500	(73.1)
≥28	24	(6.7)	22	(6.7)	46	(6.7)
**Family history**						
Yes	29	(8.1)	31	(9.5)	60	(8.8)
No	329	(91.9)	295	(90.5)	624	(91.2)
**Smoking**						
Never	126	(35.2)	109	(33.4)	235	(34.4)
Ever	232	(64.8)	217	(66.6)	449	(65.6)
**Drinking**						
Never	187	(52.2)	169	(51.8)	356	(52.0)
Ever	171	(47.8)	157	(48.2)	328	(48.0)
**Pathological type**						
Highly	62	(17.3)	65	(19.9)	127	(18.6)
Moderately	201	(56.1)	178	(54.6)	379	(55.4)
Low	95	(26.5)	83	(25.5)	178	(26)
**Stage**						
I	38	(10.6)	41	(12.6)	79	(11.5)
II	162	(45.3)	142	(43.6)	304	(44.5)
III	158	(44.1)	143	(43.8)	301	(44.0)

### Cell culture and animals

Human esophageal carcinoma cancer cell lines Eca-109 and TE-1 were purchased from the Cell Bank of Type Culture Collection of the Chinese Academy of Sciences, Shanghai Institute of Cell Biology, and were passaged for less than 6 months. Cells were maintained in RPMI-1640 medium (Gibco BRL, Gaithersburg, MD), and supplemented with 10% heat-inactivated fetal bovine serum (Gibco BRL), 2 mM L-glutamine, 100 U/mL penicillin, and 100 U/mL streptomycin. Culture plates were then incubated at 37°C in an atmosphere of 5% CO_2_.

Female BALB/c nude mice that were 4–5 weeks old were purchased from the Shanghai Laboratory Animal Center at the Chinese Academy of Sciences (Shanghai, China). All mouse experiments were performed according to the Laboratory Animal Center of Soochow University's guidance.

### Circular RNA plasmid construction

We constructed the circular RNA plasmid used in this study. Human *cir-ITCH* cDNA was synthesized by GeneWiz (Suzhou, China) and cloned into pLVX-IRES-neo (Clontech Laboratories Inc., San Francisco, CA, USA). We used a 996 bp DNA fragment corresponding to exons 6–13 of the *ITCH* gene, and added 1 kb upstream and 200 bp downstream to the nonlinear splice sites. Also, an 800-bp DNA stretch was added upstream of the splice acceptor site and inserted downstream in the reverse orientation [9]. The resulting construct (*pLVX-cir-ITCH*) was verified by direct sequencing.

### Lentiviral production and transduction

The cDNA sequence of *cir-ITCH* was synthesized by the Genewiz Company (Suzhou, China) and then cloned into the lentiviral expression vector pLVX-IRES-neo (Clontech Laboratories Inc., San Francisco, CA, USA). Lentiviral production and transduction were conducted by following previously published procedures [44].

### RNA extraction and real-time quantitative polymerase chain reaction

Total RNA was isolated from cells and tissues using the TRIzol® reagent (Invitrogen) according to the manufacturer's instructions. Esophageal tissue samples from newly diagnosed ESCC patients were immediately placed in liquid nitrogen and then stored at −80°C before analysis. cDNA was synthesized from total RNA using SuperScript III® (Invitrogen) according to the supplied protocol. The relative gene expression of *cir-ITCH* was determined using the ABI Prism 7500 sequence detection system (Applied Biosystems, Foster City, CA, USA), which is based on the TaqMan method. *GAPDH* was used as an internal standard control, and all reactions were performed in triplicate [45]. The primers used for polymerase chain reaction (PCR) amplification are listed in Table 2.

**Table 2:** The sequences of primers, probes, used in this study

Primers for PCR	Forward	Reverse	probe
*Cir-ITCH*	GCAGAGGCCAACACTGGAA	TCCTTGAAGCTGACTACGCTGAG	CCGTCCGGAACTATGAACAACAATGGCA
*GAPDH*	CAATGACCCCTTCATTGACC	TTGATTTTGGAGGGATCTCG	CTGAGAACGGGAAGCTTGTC
*Linear ITCH*	TAGACCAGAACCTCTACCTCCTG	TTAAACTGCTGCATTGCTCCTTG	
*Circular ITCH*	ACAGAGACAACCGAGAAACAGTG	GCCTTGATACTTGTTACCGTCGA	
*c-Myc*	TTCGGGTAGTGGAAAACCAG	CAGCAGCTCGAATTTCTTCC	
*GAPDH*	GAAGGTGAAGGTCGGAGTC	GAAGATGGTGATGGGATTTC	

### RNase R digestion

The RNase R digestion reaction was performed following previously published procedures. The digestion and precipitation reactions were repeated twice with a ratio of 3 U enzyme/1 mg RNA [8].

### Transient transfections and luciferase assays

Eca-109 and TE-1 cells were seeded in 24-well plates (1 × 10^5^ cells per well) and cultured to 60–70% confluence before transfection; then, cells were transfected with 800 ng of the reporter plasmids described above using Lipofectamine 2000 (Invitrogen, CA, USA). Cells were co-transfected with 0, 1 or 40 pmol of miR-216b, miR-17, miR-214, miR-7, and miR-128 mimics according to the manufacturer's instructions [46]. *cir-ITCH* hyperexpression in cells was evaluated with the same test. Each group included 6 replicates, and independent triplicate experiments were performed. After transfection for 24 h, the cells were collected using 100 μL passive buffer, and Renilla luciferase activity was detected using the Dual-Luciferase Reporter Assay System (Promega), and a TD-20/20 illuminometer (Turner Biosystems, Sunnyvale, CA); the results were normalized against the activity of the *Renilla* luciferase gene.

### Actinomycin D assay

TE-1 and Eca-109 cells were seeded at 5 × 10^4^ cells per well in 10 mm 24-multiwell plates. Sixteen hours later, the cells were transiently transfected using Lipofectamine 2000 (Invitrogen) and co-transfected with 1 or 40 pmol of miRNA mimics (Ambion) with or without 40 pmol of miRNA inhibitor, as indicated, for 24 h; then the cells were exposed to actinomycin D (Sigma, St Louis, MO) for 1, 2, and 3 h. The cells were harvested and the stability of the *cir-ITCH* mRNA was analyzed using quantitative reverse transcriptase PCR (qRT-PCR). Actinomycin D was used at concentrations of 2 mg/L.

### Western blotting

Protein lysates from ESCC tissues and cells were subjected to western blot analysis according to standard protocols as previously described [47]. Antibodies recognizing ITCH, Dvl2 and β-Actin were from Cell Signaling. Antibodies recognizing β-catenin and Wnt3a were from Abcam.

### Cell viability assay

In 96-well, flat-bottomed plates (BD Biosciences, Bedford, MA), 100 μL of Eca-109 and TE-1 cell suspensions (10,000 cells per mL) were aliquoted into each well. After transfection, as described previously for the actinomycin D assay, and 1, 2, and 3 days of cultivation, cell viability was measured by the Cell Counting Kit-8 (CCK-8) system (Dojindo Laboratory, Kumamoto, Japan) according to the manufacturer's instructions. Briefly, 10 μL of CCK-8 solution was added to each well, the plates were incubated at 37°C for 1 h, and the absorbance of each well was read at 450 nm using a microplate reader (MRX; Dynex Technologies, West Sussex, United Kingdom). There were 6 replicates for each group, and the experiments were repeated at least 3 times.

### Colony formation assay

ESCC cells with stable *cir-ITCH* overexpression and controls (80 cells) were seeded into a 60-mm plate. After incubation for 2 weeks at 37°C in a 5% humidified CO_2_ atmosphere_,_ colonies (>50 cells per colony) were stained with Giemsa, counted, and photographed. Each experiment was performed in triplicate.

### Cell cycle analysis

For cell cycle analysis, ESCC cells with stable *cir-ITCH* overexpression were stained with 0.5 mL propidium iodide (PI) staining solution, and cellular DNA content was analyzed by flow cytometry (FACScalibur; BD Biosciences).

### Xenografts in mice

Eca-109-empty vector, Eca-109-*cir-ITCH*, TE-1-empty vector, and TE-1-*cir-ITCH* cells were diluted to a concentration of 1 × 10^6^/mL in physiological saline. Mice were injected subcutaneously with 0.1 mL of the suspension into the back flank. When a tumor was palpable, tumor growth was measured every other day with a caliper. Tumor volume (V) was calculated according to the following formula: V = L × W^2^ × 0.5 (L, length; W, width).

### Statistical analyses

One-way analysis of variance and linear regression models were used to identify the effect of altered *cir-ITCH* expression on the *ITCH* mRNA levels in ESCC cells, and the correlation between the expression of *cir-ITCH* and the *ITCH* gene in ESCC tissues. Differences between groups were assessed by a paired, 2-tailed student's t-test. A *P*-value of <0.05 was considered statistically significant.

## SUPPLEMENTARY MATERIAL FIGURE AND TABLE


