# Supplementary material for: Circular RNA ITCH has inhibitory effect on ESCC by suppressing the Wnt/β-catenin pathway
Source: Oncotarget. 2015 Feb 28;6(8):6001–13. doi: 10.18632/oncotarget.3469 (PMC4467417; doi:10.18632/oncotarget.3469)
Supplement: Supplementary file 1 [file oncotarget-06-6001-s001.pdf]

Circular RNA ITCH has inhibitory effect on ESCC by suppressing the Wnt/β-catenin pathway

Supplementary Material

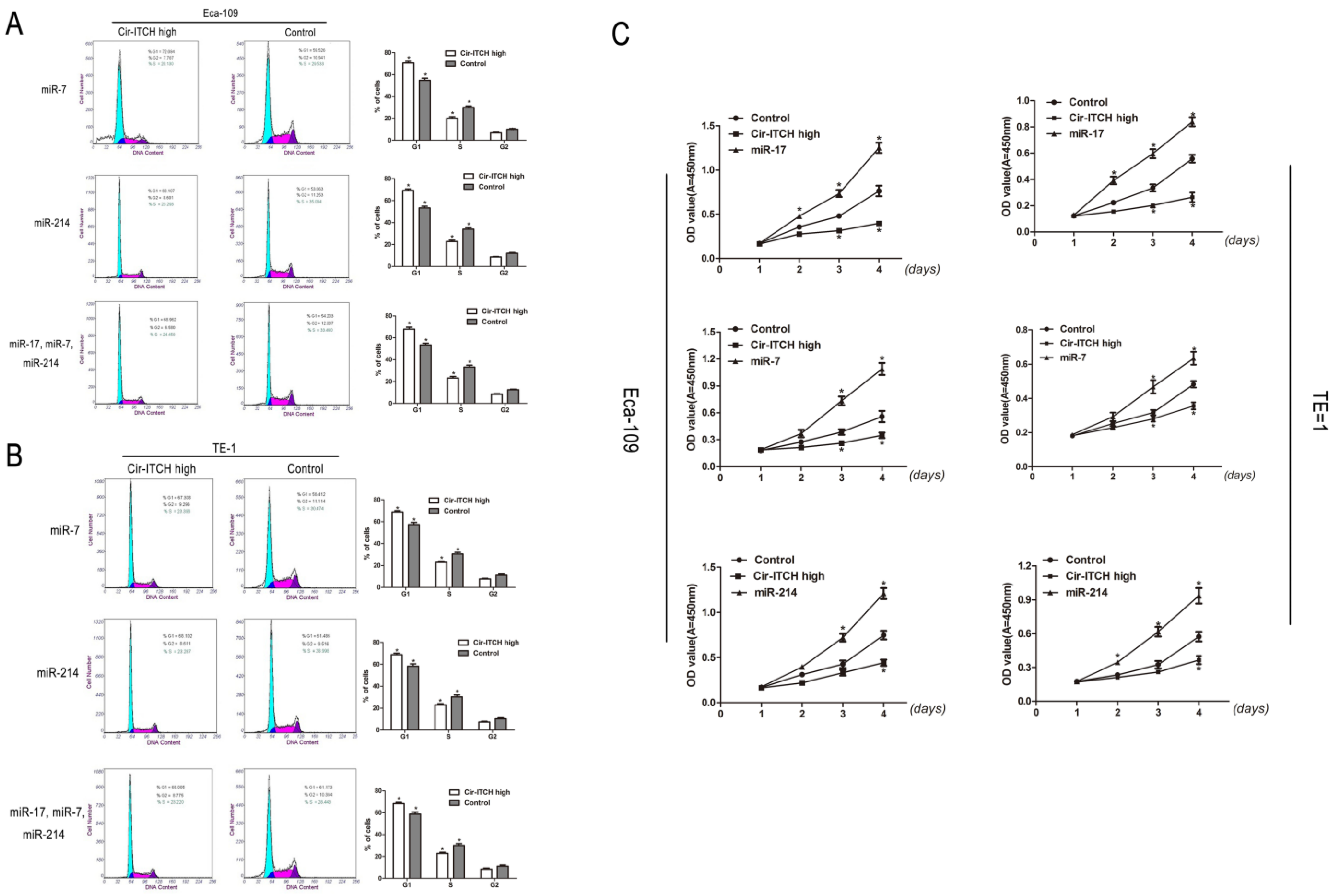

Supplementary Figure S1: The effects of ectopic *Cir-ITCH* expression on ESCC cell cycle and proliferation. (A)(B) Cell cycle analysis of Eca-109 and TE-1 cells after transfected with *Cir-ITCH* in the presence of miR-7, miR-214 and all of the miR-17, miR-7, miR-214, or Control lentiviruses. Flow cytometry data are represented as mean±SEM and are based on three independent experiments. (C) Eca-109 and TE-1 cells were seeded in 96-well plates after transfected with *Cir-ITCH* in the presence of other miRNAs and Control lentiviruses, and cell proliferation was performed daily for 3 days using the CCK-8 assay. Six replicates for each group and the experiment repeated three times. Data are mean±SEM. *P<0.05 compared with controls.

Supplementary Table S1: The sequence of the predicted miRNA binding sites on the the 3'-UTR region of *ITCH* and *cir-ITCH*

microRNA	miRNA binding sites 3'-UTR	miRNA binding sites in *cir-ITCH*
miRNA-17	cauuauuaacugauuaaugcacuuug	caaagugcuuacagugcagguag
miRNA-7	guggccacauguauaugucuuccc	ugagguaguagguuguauaguu
miRNA-214	uguauaugucuucccugcugu	acagcaggcacagacaggcagu
miRNA-216b	ccuacaauauuuacuagagauuu	aaaucucugcaggcaaauguga
miRNA-128	uacaaacaauguuaacacuguga	ucacagugaaccggucucuuu
